# Predicting the Future: Mirror Neurons Reflect the Intentions of Others

**DOI:** 10.1371/journal.pbio.0030109

**Published:** 2005-02-22

One of the more intriguing recent discoveries in brain science is the existence of “mirror neurons,” a set of neurons in the premotor area of the brain that are activated not only when performing an action oneself, but also while observing someone else perform that action. It is believed mirror neurons increase an individual's ability to understand the behaviors of others, an important skill in social species such as humans. A critical aspect of understanding the behavior of another person is recognizing the intent of his actions—is he coming to praise me or to bury me? In this issue, Marco Iacoboni and colleagues use functional magnetic resonance imaging (fMRI) to show that the mirror neuron system tracks not only the actions, but also the intentions, of others.

The researchers presented subjects with one of three types of movie clips, “context,” “action,” and “intention.” The “context” clip came in two versions. In the first, a mug of tea, a teapot, a pitcher of cream, and a plate of cookies sit neatly on a nondescript surface. In the second, the mug is empty, the pitcher is on its side, and a napkin lies crumpled beside scattered cookie crumbs—the dregs of an apparently well-enjoyed snack. The “action” clip shows only an empty mug, being grasped either by the whole hand around the rim (called whole-hand prehension), or with the fingers on the handle (precision grip). The “intention” clip puts it all together, providing the context needed to understand the intent of the action. In the first context scene, the mug is grasped in the context of a well-laid spread, signaling intent to drink the tea. In the second, the mug is grasped against the backdrop of the rest of the tea-time dregs, signaling the intent to clean up. In both cases, the type of grasp used is alternated, to avoid implying intent merely based on grip type.

The essential interpretive technique in fMRI is to subtract the activation patterns from two different stimuli, thereby highlighting brain regions that are activated differentially in response to the difference in stimulus. In this study, “intention minus context” showed areas involved in recognizing both action and intention, while “intention minus action” showed areas for both context and intention. Comparing these two results, Iacoboni and colleagues found that an area in the right inferior frontal cortex, an area known to be involved in the mirror system, was activated only by scenes in which intention could be inferred. In the authors' words, “it appears that there are sets of neurons in human inferior frontal cortex that specifically code the ‘why’ of the action and respond differently to different intentions.”

They note that these neurons differ in function from previously defined mirror neurons in that they apparently code not current actions, but some aspect of future ones. In this interpretation, an action observed within a familiar context activates mirror neurons for “logically related” actions, those that most likely will follow the observed one. This suggests the mirror neuron system is intimately involved not only with understanding the behavior of others, but predicting it as well.

